# UnderstandingDelirium.ca: A Mixed-Methods Observational Evaluation of an Internet-Based Educational Intervention for the Public and Care Partners

**DOI:** 10.3390/geriatrics10060168

**Published:** 2025-12-16

**Authors:** Randi Shen, Dima Hadid, Stephanie Ayers, Sandra Clark, Rebekah Woodburn, Roland Grad, Anthony J. Levinson

**Affiliations:** 1 Faculty of Health Sciences, McMaster University, Hamilton, ON L8S 4L8, Canada; 2 Department of Family Medicine, McGill University, Montréal, QC H3A 0G4, Canada

**Keywords:** delirium, caregiver education, e-learning, internet-based intervention, health literacy, family care partners, public health, prevention

## Abstract

**Background/Objectives:** Delirium, an acute cognitive disturbance, is often unrecognized by family or friend care partners, contributing to delayed interventions and negative health outcomes. UnderstandingDelirium.ca is an e-learning lesson developed to address this gap by improving delirium knowledge among the public, patients, and family/friend care partners. Our objective was to evaluate the acceptability, intention to use, and perceived impact of Understanding Delirium e-learning among public users. **Methods:** A convergent mixed-methods observational evaluation combining survey-based quantitative data and thematic analysis was conducted. The survey included the Net Promoter Score (NPS), the short-form Information Assessment Method for patients and consumers (IAM4all-SF), and an open-text feedback item. Descriptive statistics were used to summarize IAM4all-SF responses, assessing perceived relevance, understandability, intended use, and anticipated benefit. Open-text comments were analyzed thematically by two independent reviewers who reached consensus through discussion. Subgroup analysis of qualitative themes was performed by age, gender, and NPS category. **Results:** Among 629 survey respondents, over 90% of respondents agreed that the lesson was relevant, understandable, likely to be used, and beneficial. The NPS was rated ‘excellent’ (score of 71), and lesson uptake included over 7000 unique users with a 35% completion rate. Qualitative analysis revealed themes of high educational value, emotional resonance, and perceived gaps in prior healthcare communication. Respondents emphasized the lesson’s clarity, intent to share, and potential for wider dissemination. **Conclusions:** UnderstandingDelirium.ca is a promising, guideline-aligned digital intervention that has potential to enhance delirium literacy and reduce care partner distress. Findings suggest that the Understanding Delirium e-learning can effectively improve public delirium literacy and should be integrated into care partner and clinical workflows.

## 1. Introduction

Delirium is an acute cognitive disturbance commonly caused by underlying medical conditions, metabolic disturbances, and medical interventions. This disorder is associated with increased morbidity and mortality. In the community, delirium prevalence is estimated at 1–2% and rises with age to 14% in individuals above 85 years old [1]. In emergency department settings, delirium may be present in up to one-third of patients, depending on age and other factors, but is often unrecognized [2,3,4,5]. In hospitals 11–25% of older adults will have delirium on admission, and 29–31% will have developed delirium during their stay [6,7]. Since delirium symptoms fluctuate, and patients may have varied cognitive baselines, family/friend care partners are often best positioned to notice changes.

Family/friend care partners are often spouses, children, family members or friends who provide unpaid care to patients living with chronic illnesses. These individuals are often not formally trained or tied to any professional health organization. Family members and other care partners are interested in receiving information about delirium, including symptoms and causes, as well as what they can do to help [8,9]; however, such information is often not provided [8,10]. Evidence shows that the effectiveness of delirium detection and management improve when family care partners are engaged [11]. However, there is a limited number of evidence-based educational interventions to prepare family care partners for this role. Most existing delirium education programs are hospital-centered, rely on in-person sessions, or offer limited interactivity or accessibility for community-based care partners [8,9,10]. Enhancing delirium literacy among the public and care partners is critical for early detection and better care outcomes. Furthermore, care partners for delirium patients are at risk of increased distress and adverse psychological impacts [12,13]. Improving delirium literacy has been shown to empower care partners and reduce emotional burden [14].

**Figure 1:**
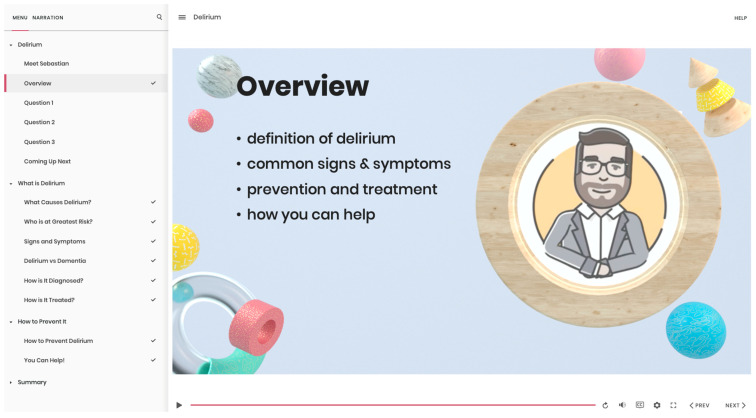
UnderstandingDelirium.ca e-learning lesson.

UnderstandingDelirium.ca is a 15 minute, asynchronous, e-learning lesson developed to address the need for improving delirium knowledge among the public and family/friend care partners (Figure 1). Figure 1 illustrates the interface and structure of the e-learning lesson. The development phase involved a collaborative process with experts in delirium care, instructional design, and patient education, as well as people with lived experience as care partners. The lesson content included delirium signs and symptoms, prevention strategies, and guidance for how care partners can help.

A user-centered approach ensured content was accessible, engaging, and culturally appropriate for diverse audiences. Best practices in multimedia learning instructional design were also used. Initial user testing took place in December 2022–January 2023 and led to improvements to the case scenario of the lesson, among other enhancements. UnderstandingDelirium.ca is hosted within the McMaster Optimal Aging Portal, a free, publicly accessible, evidence-based health information website for adults and older adults. The Portal curates high-quality health evidence in user-friendly formats (e.g., evidence summaries, blog posts, videos) and disseminates content primarily through its website and subscription e-newsletter. Previous evaluations of the Portal have demonstrated high usability and satisfaction, and have described its users as predominantly female, highly educated, and typically aged 55 years and older, many of whom self-identify as caregivers or individuals managing their own chronic conditions [15,16]. This study aims to analyze user feedback of the lesson for future improvements and to evaluate learners’ perceptions of its relevance, understandability, use, and perceived benefits.

## 2. Materials and Methods

### 2.1. Study Design

A single-group, post-intervention, mixed-methods observational evaluation using a post-lesson survey was conducted.

### 2.2. Participants and Recruitment

Potential respondents were recruited from the McMaster Optimal Aging Portal subscriber list with a link to the delirium e-learning lesson; the lesson was also made available publicly on the internet [15,16]. Eligible participants were limited to those who completed the lesson and voluntarily opted into the post-lesson survey. Incomplete survey responses were excluded from analysis.

### 2.3. Outcomes

Following completion of the lesson, users were invited to complete a brief survey. Anonymous data were collected continuously from 16 February 2023–31 January 2025 as part of an ongoing post-implementation evaluation.

Data were collected after lesson completion using the Net Promoter Score (NPS) and the short-form version of the Information Assessment Method for All Questionnaire (IAM4all-SF), both included in the post-lesson survey. A copy of the IAM4all-SF has been included in the Supplementary Materials. The NPS was used as an outcome measure of overall satisfaction and willingness to recommend the lesson. It is a single question asking respondents: “How likely is it that you would recommend the lesson to a friend or colleague?” on a scale from 0 (not at all likely) to 10 (extremely likely) [17]. The IAM4all-SF assessed perceived relevance, understandability, intention to use the information, and anticipated benefit on a 5-point Likert scale from strongly disagree (1) to strongly agree (5). It is a short-form version of the Information Assessment Method (IAM4all), a content-validated tool used to assess and improve consumer-oriented health information [18]. We created the short form to minimize response burden and improve completion rates, particularly given the older adult demographic targeted by the intervention.

Respondents were also asked to share basic demographic data like age, gender and how they were directed to the e-learning website. To reduce survey burden and to support anonymity, we did not collect additional sociodemographic details. Additionally, open text feedback was collected to allow respondents to elaborate on their answers, provide additional comments, or suggest improvements. Web and learning analytics, including pageviews, lesson starts, conversion rate, and completions were also collected.

### 2.4. Data Analysis

A convergent mixed-methods research design was used to evaluate user feedback. The use of a convergent mixed-methods approach allowed for the triangulation of quantitative user ratings with qualitative feedback to better understand the perceived impact and areas for improvement of the e-learning lesson.

#### 2.4.1. Quantitative Data Analysis

Quantitative data from the IAM4all-SF questionnaire were collected using SurveyMonkey and exported to IBM SPSS Statistics version 30 for analysis. Descriptive statistics, including frequency counts and percentages, were used to summarize participants’ responses across four domains: perceived relevance, understandability, intention to use the information, and anticipated benefit. NPS was calculated to assess users’ likelihood of recommending the lesson to others. The NPS scoring question stratifies the respondents into three categories, promoters (score 9–10), passives (score 7–8) and detractors (score 0–6). NPS is calculated using the standard scoring method by subtracting the percentage of detractors from promoters, resulting in a single score for the entire program ranging from −100 to +100 [17]. Subgroup analyses of IAM4all-SF scores and NPS ratings were conducted by age, gender, and NPS category using the Kruskal–Wallis test. Due to the small number of respondents in the younger age bands, participants under age 55 were aggregated into a single category for the purposes of subgroup analysis. This approach ensured adequate cell sizes for comparison and improved the precision of statistical estimates.

#### 2.4.2. Qualitative Data Analysis

Qualitative data were analyzed using thematic content analysis [19]. Using an inductive approach, free text comments were manually coded using Microsoft Excel (version 16.103.4) independently by two reviewers [RS + DH], with discrepancies resolved through discussions to reach consensus on the final coding framework. Analytic rigor was enhanced through formal comparisons of themes across participants and collaborative interpretation within the research team. Final themes and sub-themes were reviewed and approved by the wider research team. Subgroup analyses were conducted by age, gender and NPS category to explore thematic patterns in relation to participants’ demographics and satisfaction with the lesson. Triangulation was achieved both across analysts and across data sources.

### 2.5. Data Integration

Data integration occurred during both the design and interpretation phases. The project was structured around predefined research objectives that guided the integration of qualitative and quantitative strands [20]. After independent analysis, results from both components were merged in a narrative to provide a comprehensive understanding of the findings. For example, qualitative feedback helped explain high NPSs by revealing personal resonance and desire to share with family.

### 2.6. Ethics Approval

The Hamilton Integrated Research Ethics Board reviewed the study protocol and granted exemption from full review per their review process, as this was considered a quality improvement evaluation.

## 3. Results

Results are reported sequentially: sample and engagement, survey outcomes, and thematic analysis.

### 3.1. e-Learning Lesson Uptake

The Understanding Delirium e-learning lesson landing page was accessed by 7096 unique users, resulting in 10,794 pageviews. Of these users, 4706 initiated the lesson, corresponding to a conversion rate of 66.3%. Among those who began the lesson, 1650 individuals completed it, yielding a lesson completion rate of 35.1%.

### 3.2. Demographics

Of the 1650 individuals who completed the delirium e-learning lesson, 739 agreed to participate in the post-lesson survey, and 629 (85.1%) completed it (Table 1). Most respondents (79.8%) accessed the lesson via the McMaster Optimal Aging Portal e-newsletter. The majority of respondents were female (85.4%), and over 70% were aged 65 or older, including more than one-third who were 75 years or older.

### 3.3. Quantitative Analysis

**Figure 2:**
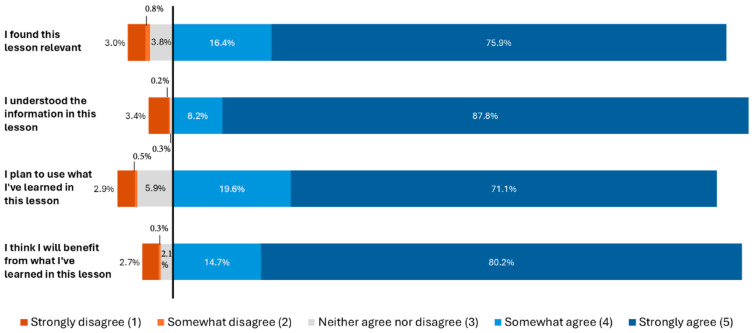
IAM4all-SF summary chart. Respondents agreed that the lesson was relevant and understandable, and that they intended to use it and anticipated benefit.

Results from the IAM4all-SF questionnaire indicated high levels of user endorsement and impact across all domains. The median and mode for each domain was 5 (strongly agree). Specifically, 579 of 627 (92%) of respondents somewhat or strongly agreed that the lesson was relevant; 599 (96%) found the information understandable; 569 (91%) intended to use what they had learned; and 594 (95%) believed they would benefit from it. The NPS was rated as ‘excellent,’ with a score of 71 on a scale from −100 to +100, indicating a very high likelihood to recommend the lesson. Detailed results are presented in Figure 2, and Table 2 and Table 3.

For analysis purposes, age groups were collapsed into four broader categories, with all respondents under 55 combined into a single group to ensure sufficient sample size for statistical testing.

A Kruskal–Wallis test indicated a statistically significant difference in NPS by age group, H(3) = 16.45, *p* < 0.001, as well as in NPS category (promoter, passive, detractor) by age group, H(3) = 14.59, *p* = 0.002. However, post hoc comparisons could not be conducted due to tied median values and insufficient valid cases in some groups. Analysis of IAM4all-SF responses by age group showed a statistically significant difference for the item related to intended use of information (H(3) = 9.86, *p* = 0.020), but not for the other IAM4all-SF domains. Similarly, significant differences in IAM4all-SF scores were observed across NPS categories, with higher ratings consistently reported among promoters. Due to the limited variability in responses and the presence of tied ranks, post hoc analyses were not feasible.

Overall, these findings indicate a high level of acceptability, likelihood to recommend, and potential for practical application among respondents.

### 3.4. Qualitative Analysis

A total of 423 respondents provided open-text comments. Of these, 62 were deemed non-contributory (e.g., blank, irrelevant, or unintelligible), resulting in 361 responses included in the qualitative analysis. Thematic content analysis identified five major themes and 20 subthemes. The five themes were: (1) educational value of the lesson; (2) personal and professional relevance; (3) suggestions for improvement; (4) emotional and psychological impact; and (5) healthcare system and professional support. These themes are summarized in Table 4 and themes, subthemes, and additional quotations are available in Supplementary Table S1.

Respondents overwhelmingly highlighted the educational value of the e-learning lesson. Many described the lesson as informative, well-structured, and clearly presented.

Respondents also highlighted how the lesson clarified previously held misconceptions by providing a clearer understanding of delirium and its distinction from dementia:

“It was helpful to understand how it is different from dementia. Dementia is such a well-discussed topic, and I’m sure so many lay people are not very familiar with delirium and simply assume their loved one is experiencing dementia.”

(ID 629)

The lesson also enabled respondents to make sense of past experiences involving delirium in loved ones. Some expressed regret at not having had access to this information earlier:

“My husband died of pancreatic cancer a year ago. I wish I’d known more about delirium then, because that knowledge would’ve helped me understand what he was going through in the weeks before his death. Now, in retrospect and with the information presented in these lessons, it all makes so much more sense. Thank you.”

(ID 529)

This retrospective clarity frequently provided respondents with a sense of relief and, in some cases, a sense of closure. Many expressed gratitude for finally understanding what had occurred during distressing experiences with loved ones:

“Almost 18 months ago I experienced delirium while in hospital. I am just now coming to terms with all the pieces this was complicated by system becoming septic twice ….actually did almost die twice. Still feel fearful…..cannot remember much of 1st year of recovery. Finally understanding what happened…and be less fearful.”

(ID 103)

While many respondents were learning about delirium for the first time, some viewed the lesson as a helpful refresher, a reinforcement of knowledge they had previously encountered, particularly among those with prior familiarity with delirium or clinical experience.

Beyond the educational content, respondents consistently praised the clarity and accessibility of the lesson format. The presentation style, visual layout, and lesson design were praised for supporting engagement and comprehension.

Respondents frequently indicated an intention to share the lesson among family members, friends, and caregiving networks, particularly those supporting older adults with dementia or individuals who had recently experienced episodes of delirium:

“I will share this lesson with family members and friends who have parents experiencing delirium.”

(ID 313)

Others expressed plans to incorporate the knowledge into caregiving practices or to apply it for proactive health monitoring, both for others and for themselves.

Several respondents reported that they were not informed about the condition during critical moments of care, which contributed to confusion and distress:

“My father experienced delirium after surgery to remove stomach cancer 10 yrs ago...No one on the team explained what was happening. The amount of stress we suffered trying to figure out what was happening was immense.”

(ID 62)

Others described instances in which their concerns were dismissed or overlooked by medical staff despite observable changes in their loved ones’ behavior.

In response to these experiences, respondents advocated for systemic improvements, including the integration of delirium education into hospital protocols, caregiving teams, and broader public health initiatives. Many emphasized that delirium awareness and training should be incorporated into standard education for both family/friend care partners and healthcare professionals. This was also echoed by professionals, including nurses and health workers, who recognized the lesson’s clinical value, with some proposing its integration into staff training and orientation programs.

While overall feedback was highly positive, respondents provided several constructive suggestions to improve the lesson. These included technical enhancements, such as incorporating autoplay functionality and streamlining transitions between sections. Others recommended expanding the content to cover additional topics, including alcohol withdrawal delirium, and more specific, actionable strategies for care partners. Suggestions also addressed accessibility and interactivity, such as adjusting font size for readability and including final knowledge checks to reinforce learning.

When analyzing responses by subgroups of gender, age, and NPS rating, minor but notable differences emerged. Female respondents more frequently referenced the lesson’s value in helping them understand prior experiences with delirium and in supporting proactive health management for others, compared to male respondents. Age-related trends were also observed: respondents under the age of 65 more often emphasized the educational and informative aspects of the lesson, while those aged 65 and older more frequently discussed its usefulness in retrospectively understanding previous experiences with delirium and in guiding proactive health management for both themselves and others. Respondents aged 75 and older were particularly likely to express intentions to share the knowledge they had gained with family and friends.

Analysis by NPS category revealed that both promoters (*n* = 271) and passives (*n* = 72) commonly mentioned the lesson’s usefulness in understanding prior experiences with delirium, raising awareness, and supporting proactive health behaviors. These groups also expressed feelings of relief and gratitude for understanding what had occurred during distressing experiences. Promoters, in particular, frequently praised the clarity and organization of the content. In contrast, detractors (*n* = 12) were more likely to highlight the need for improved professional support, particularly noting a lack of communication from healthcare providers during delirium-related experiences.

### 3.5. Integration of Quantitative and Qualitative Findings

The qualitative findings aligned closely with quantitative IAM4all-SF results and NPS ratings. Participants’ descriptions of the lesson as being clear, informative, and emotionally resonant correspond with high IAM4all-SF ratings of relevance and understandability. Similarly, repeated intentions to apply and share what was learned reflect strong agreement with the IAM4all-SF domains of intention to use and anticipated benefit, as well as the high overall NPS, indicating willingness to recommend the lesson. Comments emphasizing clarity, usability, and personal resonance offer explanatory context for these quantitative results, suggesting that both cognitive and affective responses influenced participants’ likelihood to recommend the lesson. These findings demonstrate convergence between quantitative and qualitative stands, highlighting the perceived credibility, utility, and emotional value of the UnderstandingDelirium.ca lesson.

## 4. Discussion

This study aimed to evaluate learners’ perceptions of the value and impact of the UnderstandingDelirium.ca asynchronous e-learning lesson and to gather feedback for future improvements. Overall, the quantitative analysis demonstrated a high likelihood to recommend, and strong positive ratings across all IAM4all-SF domains, with over 90% of respondents agreeing that the lesson was relevant and understandable. Furthermore, they intended to use the information, and anticipated benefit.

Although IAM4all-SF scores varied significantly by NPS category—as expected given the alignment between satisfaction and perceived benefit—other subgroup comparisons yielded few statistically significant differences. These findings should be interpreted cautiously, given the skewed distribution of responses, limited variability, and small sample sizes in some age subgroups.

However, one age-related trend that did emerge across multiple measures–namely, higher NPS ratings and greater intention to use the lesson among younger respondents–may reflect differences in caregiving context. Younger participants may be more likely to be actively involved in caregiving roles for older adults, such as parents or grandparents. As a result, they may find the content more immediately relevant and actionable, which could account for their slightly greater likelihood of intending to use and recommending the information. In contrast, older respondents may be more likely to reflect on past experiences with delirium or view the lesson as less directly applicable to their current role. Future studies should consider capturing caregiving status to better understand how care context influences perceptions of value and engagement with digital education tools.

Beyond knowledge acquisition, qualitative comments revealed an emotional impact with the intervention. Many participants described feelings of relief, understanding, and closure after recognizing prior delirium experiences in loved ones or themselves. These affective responses suggest that accessible education may mitigate care partner distress and enhance confidence during future care experiences.

Interpretation of qualitative subgroup analyses by gender, age, and NPS category should also be approached with caution due to the overrepresentation of women, older adults (aged 65+), and those who rated the lesson highly (NPS promoters). Despite this, observed differences may offer early insights into varying information needs or preferences. For example, participants aged 75 and older were more likely to report intentions to share the lesson with others, particularly family members or caregivers. This aligns with a more advocacy-oriented form of engagement, even as their quantitative responses indicated a slightly lower intent to personally use the information. Dissemination strategies that leverage this motivation, such as targeting older adults in retirement or long-term care settings, may help amplify the reach and impact of the intervention. Future research is needed to confirm these patterns in more diverse populations.

The intervention was designed primarily for family and friend care partners of older adults, as well as members of the public seeking to improve delirium literacy. Recruitment through the McMaster Optimal Aging Portal likely attracted participants already motivated to engage with health-related content [21]. Notably, several health professionals, including nurses and other clinical staff, also participated and reported finding the lesson useful as a refresher. This suggests an opportunity to develop complementary educational materials tailored to healthcare providers.

### 4.1. Comparison with Previous Research

This study evaluated the intervention using the IAM4all-SF, a short-form version of the Information Assessment Method designed to assess user responses to consumer-oriented health education. We created the short form to minimize response burden and improve completion rates, particularly in the context of a slightly longer educational intervention (a 15 minute multimedia lesson) instead of a web page or short article, which are more common health information formats to which the questionnaire has been linked. Use of the IAM4all to evaluate e-learning was informed by previous work assessing the iGeriCare.ca dementia education program. In this work, the IAM4all questionnaire was also used to assess public-facing e-learning content [22]. In both cases, the instruments demonstrated strong uptake and yielded highly positive ratings across key domains, supporting their appropriateness for evaluating web-based health education.

Two systematic reviews have examined the implementation and impact of delirium education for family care partners, identifying nine relevant studies [14,23]. These interventions typically involved printed materials or in-person psychoeducational sessions [8,9,10]. Three studies that directly assessed delirium knowledge reported significant gains, while others found improvements in caregiver confidence, satisfaction, or emotional well-being. However, randomized trials comparing delirium outcomes across intervention and control groups yielded mixed results. While some showed no effect, Martinez et al. reported a reduction in delirium incidence when family-delivered, nonpharmacologic interventions were supported through education [11]. Unlike pre-existing caregiver education studies that rely on printed or in-person formats or are not targeted at the public, our work extends the evidence base by evaluating a fully asynchronous, multimedia, web-based tool designed for both public users and family/friend care partners.

Future evaluations could incorporate validated tools such as the Caregiver Delirium Knowledge Questionnaire (CDKQ) to directly assess knowledge gains [24]. For example, Wong et al. successfully used a modified CDKQ in a quality improvement initiative to evaluate the impact of a delirium education handout [25]. Other promising instruments, such as the delirium knowledge, risk factors, and attitudes questionnaire used in recent studies from Saudi Arabia [26,27], may offer broader content coverage or greater applicability across diverse cultural contexts and could also be considered. Applying such tools in future studies of UnderstandingDelirium.ca would enable objective measurement of learning outcomes.

Overall, existing delirium education programs for care partners have shown promise but have relied primarily on in-person or paper-based formats. To our knowledge, this is the first study to evaluate an asynchronous, multimedia, internet-based delirium education intervention specifically designed for both the public and family/friend care partners. Such tools have potential for wide-scale dissemination, not only in acute care but also in outpatient and community settings, where early awareness may aid in prevention and timely recognition.

e-Learning offers key advantages, including scalability, low marginal cost, and seamless integration into existing care workflows. There is also growing evidence that digital health interventions can improve caregiver well-being by reducing anxiety and enhancing self-efficacy [28]. The emotional responses observed in this study, including relief, clarity, and gratitude, support this potential psychosocial benefit.

### 4.2. Alignment with Clinical Guidelines and Implications for Practice

Our findings are especially relevant considering the recent second edition of the American Psychiatric Association delirium guidelines [29], which call for increased involvement and education of family and friend care partners in delirium care. As Shrestha and Fick and others have shown, care partners often identify symptoms earlier than healthcare providers but lack sufficient knowledge or support [9]. Care partners not only seek education about delirium but also experience substantial psychological distress. Digital education tools may be particularly well-positioned to address both the informational and emotional needs of caregivers. Chuen et al. highlight that delirium is frequently under-documented at discharge, missing a key opportunity for caregiver education, especially during transitions of care [10]. Integrating resources into transitions such as peri-operative care, Intensive Care Unit admissions, or discharge protocols may improve care partner readiness to support patient management. Our evaluation demonstrates that a brief, user-centered, web-based lesson may help to fulfill this need, aligning care practices with guideline-recommended strategies to better engage caregivers as partners in care.

### 4.3. Limitations

This study has several limitations. First, the self-selected sample and reliance on voluntary survey participation may introduce selection bias, particularly given that most participants were recruited through the McMaster Optimal Aging Portal, a source likely to attract health-motivated individuals [21]. Generalizability to other cultural or linguistic contexts may be limited in part due to the small set of demographic variables there were collected, which prevented us from comparing respondents by level of education, professional background, or caregiving status—all of which are factors that may influence how people engage with digital health information. Additionally, only those who completed the lesson and opted into the survey were included, potentially biasing responses toward those with more positive impressions. Since the evaluation did not have a pre-test or comparison group, threats to internal validity cannot be excluded. Findings should therefore be interpreted as indicators of perceived usefulness. The study also assessed perceived, rather than objective outcomes, such as knowledge gain or behavioral change. Lastly, as a web-based intervention, UnderstandingDelirium.ca may be less accessible to individuals with limited internet access, poor bandwidth, low digital literacy, or language barriers. Future adaptations should explore multimodal formats including printed materials, translated content, and audio-based tools, to ensure equitable access for diverse caregiver populations.

### 4.4. Future Directions

Future studies should assess the lesson with larger and more diverse samples in real-world settings. Implementation science and quality improvement methodologies could support integration into healthcare workflows. Randomized controlled trials or quasi-experimental designs may also help evaluate the lesson’s impact on care partner knowledge, self-efficacy, and potentially on delirium-related outcomes such as detection, duration, and severity.

## 5. Conclusions

The UnderstandingDelirium.ca e-learning lesson has the potential to be an accessible and effective tool for improving delirium literacy among the public and family/friend care partners. Findings from this pilot evaluation support its broader integration into public health and caregiver education initiatives. Future research with more diverse populations and rigorous study designs is needed to confirm these results and to assess the intervention’s impact on additional outcomes, including delirium prevention, early recognition, and caregiver well-being.

## Figures and Tables

**Table 1:** Key demographic characteristics of survey respondents.

Variables	Responses, *n* (%)
Arrived at lesson through (*n* = 621)	
McMaster Optimal Aging Portal e-newsletter	496 (79.8)
links from friends	36 (5.8)
healthcare professional recommendation	35 (5.6)
other websites	14 (2.3)
search engine	12 (1.9)
links on social media	8 (1.3)
Gender (*n* = 619)	
Female	528 (85.4)
Male	85 (13.8)
Prefer not to say	4 (0.7)
Other	1 (0.2)
Age (*n* = 610)	
≥75 years old	216 (35.4)
65–74 years old	222 (36.4)
55–64 years old	99 (16.2)
45–54 years old	35 (5.7)
35–44 years old	22 (3.6)
25–34 years old	12 (2.0)
18–24 years old	4 (0.7)

**Table 2:** Frequency of responses to items in the IAM4all-SF questionnaire.

	Responses, *n* (%)					
	Strongly Disagree	Somewhat Disagree	Neither Agree nor Disagree	Somewhat Agree	Strongly Agree	Total
I found this lesson relevant.	19 (3.0)	5 (0.8)	24 (3.8)	103 (16.4)	476 (75.9)	627 (100)
I understood the information in this lesson.	21 (3.4)	1 (0.2)	3 (0.5)	51 (8.2)	548 (87.8)	624 (100)
I plan to use what I’ve learned in this lesson.	18 (2.9)	3 (0.5)	37 (5.9)	123 (19.6)	446 (71.1)	627 (100)
I think I will benefit from what I’ve learned in this lesson.	17 (2.7)	2 (0.3)	13 (2.1)	92 (14.7)	502 (80.2)	626 (100)

**Table 3:** IAM4all-SF mean (standard deviation), median, mode, mode frequency and interquartile range.

	Mean (SD)	Median	Mode	Frequency of the Mode (%)	Interquartile Range
I found this lesson relevant.	4.61 (0.85)	5.0	5	476 (63.2)	0
I understood the information in this lesson.	4.77 (0.78)	5.0	5	548 (72.8)	0
I plan to use what I’ve learned in this lesson.	4.56 (0.86)	5.0	5	446 (59.2)	1
I think I will benefit from what I’ve learned in this lesson.	4.69 (0.78)	5.0	5	501 (66.5)	0

**Table 4:** Themes and subthemes from the qualitative analysis of open-text comments.

Themes	Subthemes
1. Educational value of the lesson	a. Informative and educational b. Better understanding of delirium vs. dementia c. Useful to better understanding of prior experiences with delirium d. Refresher/reinforcement of knowledge e. Clarity and organization of content
2. Personal and professional relevance	a. Personal use: Intend to share lesson/knowledge dissemination b. Personal use: Awareness and proactive health management for others c. Personal use: Awareness and proactive health management for themselves d. Professional use: Awareness and proactive health management for patients/clients
3. Suggestions for improvements	a. Technical issues/suggestions b. Formatting issues/suggestions c. Content expansion/suggestions
4. Emotional and psychological impact	a. Relief, clarification and gratitude b. Anxiety, fear and sadness-induced by lesson content c. Anxiety, fear and sadness-induced by experience/recollection
5. Healthcare system and professional support	a. Need for better professional support: General b. Lack of information in the healthcare and professional support system c. Lack of communication in the healthcare and professional support system d. Missed diagnosis/ignored brought up concerns e. This lesson as candidate for training professionals and patients

## Data Availability

The data presented in this study are available upon reasonable request from the corresponding author due to privacy restrictions.

## Supplementary Materials

The following supporting information can be downloaded at: https://www.mdpi.com/article/10.3390/geriatrics10060168/s1, Table S1: Additional themes and subthemes from the qualitative analysis of open-text comments.

## Abbreviations

The following abbreviations are used in this manuscript:

IAM4all-SF	Information Assessment Method for patients and consumers short form version
IAM4all	Information Assessment Method for patients and consumers
NPS	Net Promoter Score
