# Supplementary material for: UnderstandingDelirium.ca: A Mixed-Methods Observational Evaluation of an Internet-Based Educational Intervention for the Public and Care Partners
Source: Geriatrics (Basel). 2025 Dec 16;10(6):168. doi: 10.3390/geriatrics10060168 (PMC12732656; doi:10.3390/geriatrics10060168)
Supplement: Supplementary file 1 [file geriatrics-10-00168-s001.zip › geriatrics-3933617-supplementary.pdf]

Supplementary Materials

Table S1. Additional themes and subthemes from the qualitative analysis of open-text comments.

Themes	Subthemes	Selected Quotations
1. Educational value of the lesson	a. Informative and educational<br>b. Better understanding of delirium vs dementia<br>c. Useful to better understanding of prior experiences with delirium<br>d. Refresher/reinforcement of knowledge<br>e. Clarity and organization of content	*"The lesson was informative, well-presented, and useful. I was not aware of the prevalence and will review the information to enhance my knowledge base." (ID 600)*<br><br>*"It was helpful to understand how it is different from dementia. Dementia is such a well-discussed topic, and I'm sure so many lay people are not very familiar with delirium and simply assume their loved one is experiencing dementia." (ID 629)*<br><br>*"This is part of my onboarding experience as a nurse at my local hospital. It was a good refresher on delirium, since it's something I'm likely to encounter in the general medicine unit." (ID 12)*<br><br>*"This was organized in a way that was easy to follow and offered bite-sized information about delirium." (ID 15)*
2. Personal and professional relevance	a. Personal use: Intend to share lesson/knowledge dissemination<br>b. Personal use: Awareness and proactive health management for others<br>c. Personal use: Awareness and proactive health management for themselves<br>d. Professional use: Awareness and proactive health management for	*"I will share this lesson with family members and friends who have parents experiencing delirium." (ID 313)*<br><br>*"I now know what to watch for, for both of us, and I will recommend that my husband listens to the lesson too."* (ID 64)

	patients/clients	
3. Suggestions for improvements	a. Technical issues/suggestions<br>b. Formatting issues/suggestions<br>c. Content expansion/suggestions	*"The content is great. I just found that the pause between the information was not clear if there was more to come or if I should go to next."* (ID 241)<br><br>*I would like a final activity. To encourage me to interact with the info. Another few questions* (ID 603)<br><br>*I would have appreciated suggestions of what to say and do, in the moment, when I'm with someone and they are seeing things or freaking out.* (ID 530)
4. Emotional and psychological impact	a. Relief, clarification and gratitude<br>b. Anxiety, fear and sadness - induced by lesson content<br>c. Anxiety, fear and sadness - induced by experience/recollection	*"Almost 18 months ago I experienced delirium while in hospital. I am just now coming to terms with all the pieces this was complicated by system becoming septic twice ….actually did almost die twice. Still feel fearful…..cannot remember much of 1st year of recovery. Finally understanding what happened…and be less fearful." (ID 103)*<br><br>*"My father experienced delirium after surgery to remove stomach cancer 10 yrs ago...No one on the team explained what was happening. The amount of stress we suffered trying to figure out what was happening was immense." (ID 62)*<br><br>*"My husband died of pancreatic cancer a year ago. I wish I'd known more about delirium then, because that knowledge would've helped me understand what he was going through in the weeks before his death. Now, in retrospect and with the information*

		presented in these lessons, it all makes so much more sense. Thank you." (ID529)
5. Healthcare system and professional support	a. Need for better professional support: General<br>b. Lack of information in the healthcare and professional support system<br>c. Lack of communication in the healthcare and professional support system<br>d. Missed diagnosis/ignored brought up concerns<br>e. This lesson as candidate for training professionals and patients	*"This could have helped me years ago when my mother began having bouts of delirium. I eventually realized these bouts were associated with her frequent UTIs. I encourage you to as much as possible to publicize these information modules. You are correct to state that family members often recognize early signs of delirium in loved ones. At one time, my expressions of concern were ignored when Mom was in a rehab institution following hip surgery. My concerns were rebuffed, and Mom ended up returning to the hospital for many days with a severe UTI. Thank you for all that you do."* (ID 521)<br><br>*"This is a fantastic module! I would love to use this module as part of new employee orientation (health authority). This brilliantly touches on all the important aspects of delirium from prevention through treatment and would be suitable for all new employees from Housekeeping to direct care Clinical staff."* (ID 481)
